# Predictors of SARS-CoV-2 Vaccine Uptake among Health Professionals: A Cross-Sectional Study in Ghana

**DOI:** 10.3390/vaccines11010190

**Published:** 2023-01-16

**Authors:** Abdul-Samed Mohammed, Mubarick Nungbaso Asumah, Bijaya Kumar Padhi, Abhinav Sinha, Issah Mohammed, Safayet Jamil, Osborn Antwi Boasiako, Nladobi Leman, Russell Kabir

**Affiliations:** 1 Department of Environmental and Occupational Health, School of Public Health, University for Development Studies, Northern Region, Tamale P.O. Box TL1350, Ghana; 2 Department of Global and International Health, School of Public Health, University for Development Studies, Northern Region, Tamale P.O. Box TL1350, Ghana; 3 Ghana Health Service, Kintampo Municipal Hospital, Bono East Region, Kintampo P.O. Box 192, Ghana; 4 Department of Community Medicine, School of Public Health, Postgraduate Institute of Medical Education and Research, Chandigarh 160017, India; 5 ICMR-Regional Medical Research Centre, Bhubaneswar 751023, India; 6 Health Science Education Department, Faculty of Education, University for Development Studies, Tamale, Northern Region P.O. Box TL1350, Ghana; 7 Department of Pharmacy, Khwaja Yunus Ali University, Sirajganj 6751, Bangladesh; 8 Banda District Health Directorate, Banda P.O. Box 3, Ghana; 9 Banda Ahenkro Health Centre, Banda P.O. Box 3, Ghana; 10 School of Allied Health, Anglia Ruskin University, Essex, Chelmsford CM1 1SQ, UK

**Keywords:** COVID-19, pandemic, healthcare workers, vaccination, Ghana

## Abstract

COV-2 SARs has disproportionately affected low- and middle-income countries such as Ghana, where the healthcare system was not prepared enough to provide care, drugs, and equipment. This study was carried out to assess predictors of COVID-19 vaccine acceptance among health professionals in the Bono region of Ghana. A facility-based cross-sectional study was conducted among 424 health professionals recruited through simple random sampling. Univariate and multivariate logistic regression models were utilized to identify the predictors of COVID-19 vaccine acceptance presented as an odds ratio (OR) with a 95% confidence interval (CI). All respondents had heard about the COVID-19 vaccine. The most common source of information was the media (45.8%). The proportion of health professionals who accepted the COVID-19 vaccine was 73.6%. Among those who did not take the vaccine, 64.3% were willing to take it in the future. The key predictors of taking the COVID-19 vaccine included: age 25 to 45 years (AOR = 1.96, 95% CI: 1.14–3.35), age older than 45 years (AOR = 5.30, 95% CI: 2.59–10.87), males (AOR = 4.09, 95% CI: 2.34–7.15), Christians (AOR = 3.10, 95% CI: 1.44–7.72), and at least three years of experience (AOR = 1.74, 95% CI: 1.033–2.93). Reasons for not taking vaccines included: vaccines were rapidly developed and approved (41.0%), immediate side effects (39.2%), and unforeseen future effects (37.5%). This study showed that most participants had received their first dose of COVID-19 vaccination, and most of those hesitant about the vaccine were willing to receive it in the future. This is a positive finding for policy makers since it reflects that fewer resources will be needed for behavioural change initiatives. In addition, it would present a chance to focus on minority individuals who are unwilling to take the vaccine and offer targeted community mobilisation.

## 1. Introduction

The COVID-19 pandemic has posed a significant health challenge globally. Moreover, it has disproportionately affected low- and middle-income countries such as Ghana, where the healthcare system was not prepared enough to provide care, drugs, and equipment [1]. COVID-19 has been associated with a partial stop to economic activities, increased mental health problems, gender-based violence, etc. [2,3]. However, under the leadership of institutions such as the World Health Organization (WHO), humanity has come together to contain the spread of the virus. Initially, physical distancing and contact tracking were used to contain the pandemic. Still, eventually, COVID-19 vaccines were thought to be the ultimate aid in managing the pandemic by developing herd immunity in the community [4]. This led to the development of vaccine candidates with fast-tracked trials, and the final vaccines were approved for emergency use [5].

Uptake depends on the equitable supply of logistics [6]. Although, initially, disparities in accessing vaccine access were observed, these were overcome in the later stages of the pandemic [7]. The goal of the SARS-CoV-2 Development and Access Strategy, which was created in 2020 by the Africa Center for Disease Control, is to vaccinate at least 60% of the population of Africa by 2022 to acquire immune systems [8]. As of September 2021, Africa had received a total of approximately 143 million doses of the vaccine, but only 39 million individuals, which is approximately 3% of the population of Africa, were adequately vaccinated [9]. The end of the pandemic will depend a lot on how easy it is for people to get vaccines and how willing they are to take them [10]. 

Healthcare workers were prioritised to be vaccinated due to the increased risk of contracting infection among this group [11]. In addition, healthcare workers influence and set an example for the general population to get vaccinated, as reflected by a study that showed a clear correlation between vaccination reluctance among healthcare workers and vaccine aversion among the general population [12]. A systematic review reported that there was a wide range of hesitation about being vaccinated against COVID-19 among healthcare professionals around the globe, which ranged from 4.3% to 72.0% [13]. In most studies, concerns about the safety of the vaccine, its effectiveness, and the possibility of adverse reactions were identified as the primary factors contributing to COVID-19 among healthcare personnel [14]. However, vaccine acceptance increased among healthcare workers and the general population with the enforcement of trust in existing vaccines [15]. A recent study in the Bono East Region revealed that 78.6% of healthcare workers were willing to accept the COVID-19 vaccine [16]. However, there is scattered scientific evidence on predictors of vaccine utilisation among healthcare workers in Ghana. These factors can also help to increase vaccine uptake among the general population. Therefore, this study was carried out to assess the predictors of the acceptance of the COVID-19 vaccine among health professionals in the Bono region of Ghana.

## 2. Methods and Materials

### 2.1. Study Setting

The study was carried out in Banda District in the Bono Region of Ghana. This district was created in 2012 with other districts but was formally inaugurated on 28 June 2012, with the capital being Banda Ahenkro. The district lies on latitudes 7° and 8°45′ N, longitudes 2°52′ and 0°28′ W with a total land area of 2073 square kilometres. The district is bordered to the north by the Bole District, located in the Savannah Region; to the south by the Tain District; to the east by La Cote d’Ivoire; and to the west by the Kintampo South District in the Bono East Region. According to the 2021 population and housing census, the district has a population of 28,179 people with 14,813 men and 13,133 women.

### 2.2. Study Design

A hospital-based cross-sectional study was conducted with a focus on the quantitative approach. A cross-sectional study design has the advantage of allowing researchers to examine a wide range of factors simultaneously in a specific geographic region.

### 2.3. Study Population

The study included all healthcare workers (HCWs), including clinicians, allied health professionals, auxiliary employees, and employees of the district health directorate in the Banda district.

### 2.4. Sample Size and Sampling

The Snedecor and Cochran [17] formula was used to estimate the sample size:

$$N=\frac{Z^{2}*pq}{m^{2}}$$

where *N* denotes the estimated sample size, *Z* is the z-score of a 95% confidence level equivalent to 1.96, and *p* = the proportion of vaccine acceptance, which was projected as 50%. This proportion (50%) was selected and used because it gives the largest sample size with the formula. *q* = estimated vaccine hesitancy (1 − *p* = study error 0.5), and *m* = margin = 5% = 0.05 in this study.

$$N=\frac{{\left(1.96\right)}^{2}*0.5\left(1-0.5\right)}{{\left(0.05\right)}^{2}}=384.16$$

Adding a 10% non-response rate, the sample size was estimated to be 424. Therefore, the total number of HCWs that were considered the minimum representative of the population was 424. 

A simple replacement random sampling method was used to reach the ultimate sampling unit. To do this, we obtained a list of all staff under the directorate through the District Director of Health, which was generated in an excel file. Using the randomisation function in Microsoft excel, the first 424 generated numbers were considered as the selected sample. However, those who refused to participate or were transferred to other regions but still had their records captured in the district were replaced. The replacement was taken from the 425th, 426th … 449th person on the randomised Excel spreadsheet.

### 2.5. Data Collection Tools and Techniques

Data were collected using a structured questionnaire. The questionnaire was derived from previous research [16,18,19]. Because most respondents could read and write in English, the questionnaire was given to the participants to fill out and return to the researchers. The questionnaire was structured into three sections according to the study’s objectives. Section A included variables on the basic characteristics of the participants. Section B included variables on the knowledge of COVID-19 vaccines, and Section C included variables on the acceptance of the COVID-19 vaccine among HCWs.

Following randomisation, the respondents were first contacted by phone to obtain verbal consent. The questions were digitised using the Kobo collect toolbox. The respondents had the option to answer the questions via the link. Respondents were allowed to request the KoboCollect link through WhatsApp. Those without smartphones asked the link via email. The questionnaire was developed to ensure that no one submitted more than one response. Those who were in areas with poor internet networks received hard copies to fill out the questionnaire and submitted them to the researchers in an enclosed envelope. 

For the piloting and pre-test stages, a total of twenty-five HCWs in the Bole-Bamboi area were recruited. In order to get the best answers from the research participants, the questionnaire was modified based on the results of the piloting and pre-testing. 

### 2.6. Data Management, Analysis, and Presentation of Results

Adequate plans were put in place to ensure that the collected data were entered into Microsoft excel to ensure greater accuracy. Before the data were entered, all questionnaires were checked for completeness. After data entry was completed, the data were cleaned in Microsoft excel and imported to Stata version 14 (Stata Corp, Texas) for the formal analysis of the data. Analysis was conducted on two levels: descriptive and inferential analysis. The output was displayed in tables as frequencies and percentages. A univariate and multivariate logistic regression model was used to identify promoters and barriers to vaccine acceptance among health workers. This model uses the Odds Ratio estimator to determine the outcomes of each predictor variable on the dependent variable (vaccine acceptance). A *p*-value less than 0.05 was considered to be statistically significant.

### 2.7. Ethical Approval

Permission was obtained from the District Director of Health. Each respondent expressed their consent before participating in the study. Participants completed written informed consent after providing oral consent and after obtaining all the study information. The study participants were made aware that participating was completely voluntary and that they could withdraw at any time if they chose to. 

## 3. Results

### 3.1. Basic Characteristics of the Respondents

The study showed that most respondents (52.4%) were women, 55.7% had at most three years of work experience, and 88.7% were Christians. Almost half (46.7%) of the respondents were under 25 to 45 years, 60.6% were married, and 41.7% had at least a degree as their highest educational status. Most health workers (96.2%) had previously taken some form of the vaccine (Table 1).

### 3.2. Knowledge about the COVID-19 Vaccine

All respondents had heard about the COVID-19 vaccine. Only 37.3% knew that the COVID-19 vaccine was mandatory. The most common source of information was the media (45.8%). The study also showed that the following percentages of participants knew the following groups were eligible for the COVID-19 vaccine: persons under 18 years (16.0%), pregnant and lactating mothers (3.8%), people with chronic diseases (67.7%), and immunosuppressed patients (53.1%) (Table 2).

### 3.3. Acceptance and Hesitancy of the COVID-19 Vaccine

The majority of respondents (73.6%) had taken the first dose of the COVID-19 vaccine. Among those who had not taken the shot, 64.3% of them were willing to take the vaccine in the future. The fact that the COVID-19 vaccine is free (82.7%), has sufficient efficacy and safety (75.6%), protects against the virus (70.8%), is a social responsibility (61.9%), has benefits that outweigh the dangers (72.1%), and does not harm people (62.2%) and seeing others taking it (62.2%) were the reasons that motivated respondents to take the vaccine. The reasons for not taking the vaccines included: the fact that the vaccines were rapidly developed and approved (41.0%), immediate side effects (39.2%), and unforeseen future effects (37.5%). Only 24.5% of the respondents knew health workers who refused to take the COVID-19 vaccine (Table 3). 

### 3.4. Factors Influencing the Acceptance of the First Shot of COVID-19

Table 4 shows the results of both the univariate and multivariate logistic regression analyses, with the former reported in the crude odds ratio (COR) and the latter in the adjusted odds ratio (AOR) columns. First, the study showed that respondents aged 25 to 45 were 1.96 times more likely to accept the COVID-19 vaccine than those aged less than 25 years (AOR = 1.96, 95% CI: 1.14–3.35). Those over 45 years of age were 5.3 times more likely to accept the vaccines compared to those under 25 years (AOR = 5.30, 95% CI: 2.59–10.87).

In addition, males were 4.09 times more likely to accept the COVID-19 shot than females (AOR = 4.09, 95% CI: 2.34–7.15).

Respondents who were Christians were 3.10 more likely to accept the COVID-19 vaccine compared to those who were Muslims (AOR = 3.10, 95% CI: 1.44–6.72).

Respondents with at least 3 years of experience were 1.74 times more likely to accept the COVID-19 vaccine than those with less than three years of experience (AOR = 1.74, 95% CI 1.03–2.93).

## 4. Discussion

The unprecedented COVID-19 pandemic claimed many lives, which could have been prevented through vaccinations to build immunity among community members [20]. A higher proportion of vaccination means the faster development of herd immunity and, hence, better protection against infection. However, the pandemic showed a mixed response from the community towards immunisation against COVID-19, which may derail our efforts to contain the pandemic [21]. Therefore, this study was conducted to assess the factors responsible for vaccine acceptance and hesitancy in Ghana. It was observed that all participants had heard about the COVID-19 vaccine, while only one-third knew that it was mandatory. The primary source of information on COVID-19 vaccines was the media. Most participants had taken the first dose of the vaccine and were willing to take the vaccine in the future. One-quarter of the participants reported that they knew of a healthcare provider who refused to take the COVID-19 vaccine. Most participants perceived that the vaccine delivery without any cost was the main reason for taking the shot. However, the primary concern was the rapid development and approval of the vaccine for emergency use. According to the participants, community mobilisation evolved as the main way to improve the acceptance of the COVID-19 vaccine. The factors that influenced the approval of the first shot of the COVID-19 vaccine were older age, male gender, religion, higher education, and more experience. 

The study showed that all participants had heard about the COVID-19 vaccine, which reflects the importance of infection. This is in congruence with the findings of a similar study conducted among the adult population, where only around 20% of the study participants reported that it was unlikely they would be vaccinated [22]. Additionally, knowledge of COVID-19 vaccines could be used as a window of opportunity for higher vaccination coverage; since people already know about the vaccine, a little effort regarding information, education, and communication (IEC) can help increase the vaccination coverage. Furthermore, the main source of information about the vaccine for most participants was the media, highlighting that IEC activities could be carried out by using the media. The media can act as a positive influencer in spreading knowledge and breaking the myths around the COVID-19 vaccine. Moreover, a study conducted among rural communities in Wassa Amenfi in Central Ghana suggested that the media should help reduce vaccine hesitancy by decreasing the negative antecedents such as fear and less trust in leadership, which could be useful [23]. 

Furthermore, we observed that most participants had taken the first shot of the COVID-19 vaccine and were willing to take the vaccine in the future, which is consistent with the findings of a study carried out among healthcare professionals in which vaccine acceptance was reported to be high (78.6%) [16]. This is an important finding for programme managers as it highlights that the community is aware of the importance of vaccination. Here, it should be noted that resources for IEC activities could be diverted towards the relatively smaller proportion of people who need more awareness in understanding the importance of the COVID-19 vaccine. Most of the participants reported that the free vaccination provided by the government was a major reason to accept vaccines, which is in congruence with the findings of a web-based study which showed that only 55% of the study participants were willing to pay for the COVID-19 vaccine [24]. However, free vaccines hold importance in relation to global vaccine equity, as wealthier countries could quickly obtain vaccines. In contrast, low- and middle-income countries (LMICs) such as Ghana need support from vaccine manufacturers and other partners for the timely and adequate supply of vaccines [7,25]. However, efforts have been made to provide vaccines to every individual in the country [26].

A major concern amongst the study population was about the safety of vaccination, as the COVID-19 vaccines were rapidly developed and approved for emergency use. However, recent data show that most of the current COVID-19 vaccines are effective and safe and should be communicated to the masses through health education and media [27]. In addition, community mobilisation can be a strong tool to improve the acceptance of vaccines. Community engagement and participation (CEI) is an effective way to increase vaccine acceptance, which could be adopted among communities still reluctant to take shots [28].

Furthermore, we evaluated the factors that influence the acceptance of the first dose of the COVID-19 vaccine. We observed that the older age group had a higher probability of taking COVID-19 shots than the younger age group, which is similar to the reports of a mixed-method study carried out which showed that participants aged 60 years and older showed high willingness and trust in taking vaccines [8]. Higher age groups, especially >45 years, perceive that they are more prone to infection due to underlying comorbidities. Previous evidence also suggests that the chances of mortality are higher among the ageing population and amongst those who have other chronic conditions such as hypertension, diabetes, etc. [29]. However, the younger age group is no less at risk and should be aware of the benefits of taking vaccines. If this age group is infected, they may end up losing productive days, causing more economic losses. 

We observed that males had a higher likelihood of taking their first shot of the vaccine than their female counterparts, which could be due to long queues in vaccination centres, the fear of side effects, a shortage of vaccines, and misconceptions about the vaccine, as reported by a qualitative study conducted among women in Ghana [30]. This points to the need to educate females regarding the need for vaccines. Furthermore, religious influence in vaccination has been observed since Christians have a higher likelihood of taking the vaccine, which is in congruence with a review of 12 Sub-Saharan African countries, including Ghana, which also stated that vaccine hesitancy has been due to religious beliefs, which could be overcome through communication strategies by addressing the concerns of the community [31]. We observed that there were higher odds of taking the first shot of the vaccine among participants with three years of experience, which is consistent with the findings of a study that reported that occupation influenced the acceptance of the COVID-19 vaccine in Ghana [32]. 

### Implications for Policy and Practice

This study highlights that the knowledge of COVID-19 vaccines is fair among healthcare workers, which will be a strength for programme managers, as these healthcare workers are responsible for creating awareness among the masses. Moreover, a high proportion of healthcare workers are willing to accept the vaccine, which will also provide motivation and present role models for the general public. However, communities hesitant to take up vaccines need to be aware about the uses of vaccines. This could be done through community involvement and engagement processes which imply the use of a bottom-up approach, i.e., healthcare workers from their own community who have taken the vaccine may communicate with local people through a bottom-up approach. Women in older age groups should be prioritised for vaccination. Future studies should focus on predictors of COVID-19 vaccination among the general population. 

Limitations: This was a cross-sectional study among healthcare workers with representative sample size and randomisation, which implies that the findings are more generalisable. However, we did not include the general population, which is a limitation of the study.

## 5. Conclusions

This study highlights that most of the participants had taken the first shot of the COVID-19 vaccine and were willing to also take it in the future, which is a positive finding for policymakers, as less resources would be required for IEC activities. This would also give an opportunity to identify and provide focused community mobilisation and communication about behavioural changes for the small group of people who are not willing to take the shot.

## Figures and Tables

**Table 1:** Basic characteristics of respondents (N = 424).

Variables	Categories	Frequency	Percentage
Gender			
	Male	202	47.8
	Female	222	52.4
Age	<25 years	107	25.2
	25–45 years	198	46.7
	>45 years	119	28.1
Marital status			
	Single	167	39.4
	Married	257	60.6
Religion			
	Christians	376	88.7
	Islam	48	11.3
Educational qualification			
	Certificate	144	34
	Diploma	103	24.3
	Degree & above	177	41.7
Years of experience			
	≤3 years	236	55.7
	>3 years	188	44.3
Ever had any form of vaccination before			
	Yes	408	96.2
	No	16	3.8

**Table 2:** Knowledge about COVID-19 vaccine (N = 424).

Variables	Categories	Frequency	Percentage
Heard about COVID-19 vaccine			
	Yes	424	100
COVID-19 vaccine mandatory			
	Yes	158	37.3
	No	266	62.7
Who is eligible to take COVID-19 vaccine			
	Persons less than 18 years	68	16
	Pregnant and lactating mothers	16	3.8
	Persons with chronic disease	287	67.7
	Immunocompromised patients	225	53.1
Primary source of COVID-19 information			
	Workshop	178	42
	Media	194	45.8
	Others	52	12.2

**Table 3:** COVID-19 vaccine acceptance and hesitancy (N = 424).

Variables	Categories	Frequency	Percentage
Taken the first shot of the COVID-19 vaccine			
	Yes	312	73.6
	No	112	26.4
Will you consider taking COVID-19 vaccine in future (N = 112)			
	Yes	72	64.3
	No	50	44.6
Do you know a health staff who has refused the COVID-19 vaccine			
	Yes	104	24.5
	No	320	75.5
Reasons for taking the COVID-19 vaccine (N = 312) *			
	Taking the COVID-19 vaccine has no harm	194	62.2
	It protects me against the infection	221	70.8
	The COVID-19 vaccine is free	258	82.7
	Benefits outweigh dangers	225	72.1
	It is a societal responsibility	193	61.9
	Efficacy and safety sufficient	236	75.6
	Others are taking it	194	62.2
Concerns about the COVID-19 vaccines *			
	Rapidly developed and approved	174	41.0
	Unforeseen future effects not clear	159	37.5
	To promote commercial gains	111	26.2
	Immediate side effects	166	39.2
	The vaccine might be fake	73	17.2
Ways to improve COVID-19 vaccine acceptance *			
	Health education	298	70.3
	Alert and SMS reminder	238	56.1
	Community mobilisation	307	72.4

* Multiple responses.

**Table 4:** Factors influencing the acceptance of the first shot of COVID-19.

Variables	Categories	Taken First Shot of the COVID-19 Vaccine			
		COR ^a^	*p* Value	AOR ^b^	*p* Value
Age	<25 years	1		1	
	25–45 years	1.64 (0.99–2.69)	*p* = 0.051	1.96 (1.14–3.35)	*p* = 0.014
	>45 years	4.85 (2.46–9.56)	*p* < 0.001	5.30 (2.59–10.87)	*p* < 0.001
Gender	Female	1		1	
	Male	2.99 (1.88–4.77)	*p* < 0.001	4.09 (2.34–7.15)	*p* < 0.001
Marital status					
	Single	1		1	
	Married	0.99(0.63–1.55)	*p* = 0.980	1.29 (0.78–2.13)	*p* = 0.323
Religion					
	Islam	1		1	
	Christians	1.46 (0.77–2.77)	*p* = 0.250	3.10 (1.44–6.72)	*p* < 0.004
Qualification of respondents					
	Certificate	1		1	
	Diploma	1.04 (0.59–1.80)	*p* = 0.901	1.21 (0.65–2.23)	*p* = 0.547
	Degree and above	1.51 (0.91–2.49)	*p* = 0.111	1.35 (0.77–2.39)	*p* = 0.298
Years of experience					
	≥3 years	1.20 (0.77–1.86)	*p* =0.417	1.74 (1.03–2.93)	*p* = 0.037
	<3 years	1		1	
COVID-19 vaccine mandatory					
	Yes	1.04 (0.66–1.63)	*p* = 0.867	1.15 (0.70–1.89)	*p* = 0.579
	No	1		1	

R^2^ = 0.72, COR- crude odd ratio, AOR-adjusted odd ratio, ^a^—univariate logistic regression analysis, ^b^—multivariate logistic regression analysis.

## Data Availability

The data used to support this study are available from the corresponding author on request (nungbaso.asumah@uds.edu.gh).
